# Influences of gaseous substrate composition on aerobic gas fermentation by *Cupriavidus necator*

**DOI:** 10.1007/s00253-026-13990-z

**Published:** 2026-08-12

**Authors:** Brandon C. Enalls, Michael J. Wheadon, Jamia Shepard, Dagmar Leary, Jaimee R. Compton, Martin H. Moore, Satchithanandam Eswaranandam, Nicole F. Farhardi, Judson Hervey, Matthew D. Yates

**Affiliations:** 1 Center for BioMolecular Science and Engineering, US Naval Research Laboratory, 4555 Overlook Ave SW, Washington, DC 20375 USA; 2 US Army DEVCOM Soldier Center, Combat Feeding Division, 10 General Greene Ave, Natick, MA 01760 USA

**Keywords:** Gas fermentation, *Cupriavidus necator*, Single-cell protein, Poly-3-hydroxybutyrate

## Abstract

**Abstract:**

Microbial gas fermentation is a promising biotechnology for the production of a variety of industrially relevant chemicals using ubiquitous gaseous feedstocks. Implementation of efficient fermentation processes requires an understanding of the microbial response to different ratios of the gaseous feedstocks (hydrogen, carbon dioxide, oxygen) supplied to the reactor. Here, we studied the effect of inlet gas composition on the characteristics of *Cupriavidus necator*, a common strain used in industrial biotechnology due to its metabolic diversity, genetic tractability, and ability to grow to high cell densities during aerobic fermentation. We investigated whether the supply of different proportions of hydrogen and carbon dioxide in the inlet gas stream (8:1, 3:1, and 1:1 H_2_:CO_2_) while maintaining 13% dissolved oxygen affects the physiology of *C. necator*, with a focus on the accumulation of poly-3-hydroxybutyrate (PHB) and single-cell protein (SCP) within the microbial biomass. Biomass and PHB production were comparable between the 8:1 ratio (22 ± 5 g/L and 10.0 ± 0.4 g/L, respectively) and 3:1 ratio (18 ± 3 g/L and 5.8 ± 3.4 g/L, respectively) after seven days of incubation. Growth and PHB production were negatively impacted using the 1:1 ratio (8 ± 3 and 0.36 ± 1.08 g/L, respectively). SCP production varied as a function of both length of incubation and gas ratio supplied. We performed proteome profiling across time and treatment groups to assess relationships between productivity and protein expression. This study highlights the importance of gaseous substrate composition and its potential effects on microbial physiology and metabolism during fermentation.

**Key points:**

• *H*_2_*:CO*_2_* ratios of 8:1 and 3:1 produced comparable quantities of fermentation products.*

• *Essential amino acids were enriched in SCP collected from the 1:1 fermentations.*

• *Proteomics revealed differential expression in metabolic pathways of interest.*

**Supplementary Information:**

The online version contains supplementary material available at https://doi.org/10.1007/s00253-026-13990-z.

## Introduction

The reduction of single carbon (C1) compounds using hydrogen gas remains a popular method for the production of industrially relevant chemicals. This process has traditionally been accomplished using the abiotic Fischer–Tropsch Process (FTP), which converts hydrogen and carbon monoxide into liquid hydrocarbons through a series of chemical reactions (Van Der Laan & Beenackers 1999). While FTP has been widely adapted in many settings, it is resource intensive, requiring the use of high temperatures, pressures, metal-rich catalysts, as well as specific ratios of gaseous substrates (Dry 2002). The CO needed for FTP is primarily derived using a thermochemical process, such as the reverse water gas shift reaction, adding additional complexity to the production of chemicals when CO_2_ is the primary C1 feedstock available. As such, FTP requires both specialized infrastructure and pre-processed purified gaseous feedstocks for use. Gas fermentation is a promising alternative to FTP, where microorganisms are used as biocatalysts to synthesize products using hydrogen and C1 compounds as substrates. These biology-based processes are not subject to the same infrastructure and feedstock limitations as FTP, and many gas fermenting microorganisms operate at or near ambient temperature and pressure while being less susceptible to poisoning by sulfur, chlorine, and in many cases, oxygen (Michael et al. 2011; Mohammadi et al. 2011). In addition, microbial metabolisms are flexible in that, depending on the organism used, they can operate under a variety of gaseous substrate compositions (including mixes of hydrogen, carbon dioxide, carbon monoxide, methane, and/or oxygen) and can be tailored to produce a wide variety of products of industrial interest, including, but not limited, to alcohols, fatty acids, proteins, and carbohydrates (Liew et al. 2016; Woern & Grossmann 2023). While there are drawbacks to these biology-based processes (including but not limited to production rates being generally lower than those of abiotic processes and the dilution of some products into aqueous solutions as they are excreted from cells), gas fermentation has been successfully implemented at the commercial scale by companies such as LanzaTech, generating products such as ethanol from substrates such as CO, H_2_ and waste gas streams from other industrial processes (Handler et al. 2016). The continued development of these technologies is critical for the efficient transformation of these gaseous substrates, especially in instances where the desired products are accumulated within cell biomass rather than diluted into solution upon excretion.

*Cupriavidus necator* is a soil microbe whose wide metabolic flexibility makes it an ideal organism for fermentative biotechnologies. *C. necator* can grow aerobically or anaerobically using oxygen or nitrate as electron acceptors, respectively, and can grow heterotrophically on a number of organic carbon substrates or fix CO_2_ autotrophically using the Calvin Cycle via H_2_ oxidation (Cramm 2008; Johnson & Stanier 1971; Schlegel et al. 1961). *C. necator* is capable of growing to high cell densities in stirred tank bioreactors under fermentative conditions (Repaske & Mayer 1976), making it a promising candidate for the biomanufacturing of chemicals using gas fermentation. Soon after its initial description decades ago (Schlegel et al. 1961), *C. necator* had been investigated as a possible source of single-cell protein (SCP) for nutritional applications, where SCP had been shown to reach up to 74% of the organism’s biomass (Foster & Litchfield 1964). Since then, the majority of work using *C. necator* has focused on its ability to accumulate high quantities of poly-hydroxyalkanoates, including poly-3-hydroxybutyrate (PHB), as intracellular carbon storage compounds, which can then be harvested and used as precursors for bioplastic production (Zhang et al. 2022)). *C. necator* is also genetically tractable, making it a popular chassis organism for the biomanufacturing of a variety of other products (Pan et al. 2021). Given *C. necator*’s wide metabolic and physiological flexibility, there is a need to further explore and optimize autotrophic growth conditions. For example, while there are many studies examining *C. necator* physiology during gas fermentation, there are relatively few that compare the effects that changing the composition of gaseous feedstocks has on growth and productivity. Studies have reported positive correlations with biomass production and CO_2_ concentration, but have not investigated headspace compositions where CO_2_ partial pressures exceeded 5% (Flamholz et al. 2022; Panich et al. 2024). Other work has shown decreased growth and little to no PHB production by *C. necator* when H_2_ partial pressures dropped from 40 to 10% (Santin et al. 2024). The presence of CO in the gaseous substrate mix can lead to adverse effects on biomass production, where higher relative proportions of CO systematically resulted in lower biomass production. Overall SCP content within the biomass produced during CO exposure remained similar (~ 50% of dried cell weight), regardless of CO concentration present in the headspace (Jiang et al. 2022). These aforementioned studies were performed using serum bottles, where gas transfer rates are much lower than the stirred tank reactors typically used in biomanufacturing processes. Prior work using stirred tank reactors reported PHB accumulation from 58 to 79% of total *C. necator* cell mass in response to changes in O_2_ availability by adjusting O_2_ delivery methods and reactor DO concentration setpoints (Lambauer et al. 2023; Lambauer & Kratzer 2022). It remains less understood whether varying the presence of both H_2_ and CO_2_ can also affect *C. necator*’s physiology with respect to SCP and PHB production in stirred tank reactor settings.

In the present study, we conducted a series of experiments to determine whether changing the ratio of H_2_ and CO_2_ supplied to *Cupriavidus necator* in 1 L stirred tank reactors impacts cell growth as well as PHB and SCP contents within the produced biomass. Understanding these effects is important for gas fermentation technology as process economics will be significantly impacted by the amount of hydrogen that needs to be produced to maintain an optimal production rate. We coupled our growth and productivity data to a proteomics analysis to better understand changes in *C. necator*’s physiological state to determine whether fermentation productivity could be correlated to changes in intracellular biochemistry. Given that there is typically an inverse relationship between PHB accumulation and SCP production in *C. necator*, results from this study contribute understanding how feedstock composition affects the partitioning of intracellular carbon and overall performance during gas fermentation.

## Materials and methods

### Strain acquisition, media preparation, and seed culture generation

*Cupriavidus necator* strain ATCC 17699/H16/DSM 428/337 was purchased from the American Type Culture Collection (ATCC, Manassas, VA) and was routinely cultivated at 30 °C on Reasoner’s 2 A (R2A) Medium, which contains per liter: 0.5 g casein hydrolysate, 0.5 g dextrose, 0.5 g soluble starch, 0.5 g yeast extract, 0.3 g potassium phosphate dibasic, 0.3 g sodium pyruvate, 0.25 g casein peptone, 0.25 g meat peptone, 0.024 g magnesium sulfate, and 15 g agar (used in solid medium only).

Fermentation experiments were performed using a modified version of defined minimal medium DSM 55 (https://bacmedia.dsmz.de/medium/55), which contained per liter: 3.54 g sodium phosphate dibasic, 1.5 g potassium phosphate monobasic, 4.0 g ammonium chloride, 0.2 g magnesium sulfate heptahydrate, 0.01 g calcium chloride dihydrate, 0.005 g ammonium ferric citrate, 10 mL Trace element solution SL-6, and 10 mL Wolin’s vitamin solution. Trace Element Solution SL-6 contains per liter: 0.1 g zinc sulfate heptahydrate, 0.03 g manganese chloride tetrahydrate, 0.3 g boric acid, 0.2 g cobalt chloride hexahydrate, 0.01 g copper chloride dihydrate, 0.02 g nickel chloride hexahydrate, and 0.03 g sodium molybdate dihydrate. Wolin’s vitamin solution contains per liter: 2.0 mg biotin, 2.0 mg folic acid, 10.0 mg pyridoxine–HCl, 5.0 mg, thiamine-HCl, 5.0 mg riboflavin, 5.0 mg Ca-D-pantothenate, 0.1 mg vitamin B12, 5.0 mg p-aminobenzoic acid, 5.0 mg α-lipoic acid.

Single *C. necator* colonies grown on solid R2A medium were picked and inoculated into 10 mL of the defined minimal medium (with 5 g/L fructose added) in autoclaved 50 mL flasks and incubated at 30 °C overnight at 180 RPM until optical density (OD600) > 1.0) was reached. Turbid cultures were then subcultured into increasingly larger volumes until 250 mL “seed cultures” in 500 mL flasks had also reached an OD600 > 1.0, indicating readiness for inoculation into a fermenter.

### Fermentation experiment preparation

Experiments testing the effects of different gas compositions and *C. necator* strains on fermentation productivity were performed using BioXplorer 400 fermenters (H.E.L. Group, Hemel Hempstead, England) with a 1 L total volume and 700 mL working volume. Prior to the start of fermentation experiments, vessels were filled with 90% of the working volume with defined minimal medium (with no fructose or vitamin solution added) and autoclaved along with tubing used for gas supply, liquid supply, culture inoculation, and sampling. Upon cooling, 7 mL of vitamin solution was added to each vessel prior to connection to the fermenter controller that allows for automatic regulation of incubation temperature, agitation rate, pH, dissolved oxygen, and gas flow rate. Mass flow controllers built into the fermenter controller regulate the flow rate of all gases to the individual fermenters. All gases were sterilized via 0.2 μm filters just before entering the fermentation vessel. H_2_ and O_2_ were supplied in separate lines and did not mix before entering the reactor medium. Dissolved oxygen (DO) and pH were measured with in-line probes, which were also autoclaved in place with the vessels. Foaming was mitigated through the addition of Antifoam 204 in batches of 240 μL just before the start of an experiment and then once per day thereafter. The vessels were allowed to stabilize for at least 30 min after initialization under the desired experimental conditions prior to inoculation with seed cultures at 10% v/v. The same seed culture was used to inoculate triplicate fermenters for each experiment.

### Gas composition experiments

Fermentations were conducted using three different ratios of H_2_ and CO_2_ gases (8:1, 3:1, 1:1 H_2_:CO_2_) with total combined flow rate equaling 450 mL/min. Air flow rate was initially set at 30 mL/min for all experiments. The fermenter controller was programmed to increase the air flow rate upon dropping below a threshold of 13% DO saturation within the reactor, accounting for increased oxygen demand by growing *C. necator* cultures during incubations. Agitation was held constant at 250 RPM for all incubations. Medium pH was maintained at 6.5 with automatic addition of 1 M sodium hydroxide. Upon stabilization at the desired starting conditions, the vessels were inoculated with a *C. necator* H16 seed culture (at 10% v/v) and fermentation experiments ran for seven days. Samples were taken daily for immediate and downstream analyses as described below.

### Fermentation vessel sampling and preservation

Immediately after inoculation and once per day thereafter, approximately 20 mL of culture was sampled for immediate use or stored for downstream analyses. Several 1 mL aliquots of the culture were centrifuged at 5000 g for 5 min at room temperature and the supernatants were transferred to new tubes. Cell pellets were flash frozen in liquid nitrogen and stored at −80 °C for downstream protein and PHB quantification, as well as proteomics analyses. The supernatants were also flash frozen and stored at −80 °C for total nitrogen quantification.

### Biomass analysis

Cell biomass, in the form of dried cell weight (DCW) was calculated by adding 5 mL of culture to pre-weighed ceramic crucibles, drying the culture in a muffle furnace at 105 °C for 24 h, and remeasuring the weights of the crucibles containing the dried material. We also dried uninoculated minimal medium using the same method to obtain the mass of the medium components. The total biomass of the biological components of the culture was calculated by subtracting the weight of the medium components from the weight of the dried cell culture.

### Total nitrogen analyses

Frozen culture supernatants at each sample time point were thawed on ice and aliquots were diluted 1:100 in MilliQ H_2_O to quantify the total nitrogen remaining in the samples using the Simplified TKN (s-TKN) Assay (Hach, Loveland, CO) following the manufacturer’s protocol.

### Poly-3-hydroxybutyrate quantification

Poly-3-hydroxybutyrate (PHB) was quantified using methods adapted from previously described techniques (Liang et al. 2022). Briefly, frozen *C. necator* cell pellets from all time points were thawed and resuspended in 2 mL chloroform and 2 mL acidified methanol (containing 3% sulfuric acid). The resuspensions were transferred to a sealed test tube, incubated at 105 °C for 120 min, and allowed to cool to room temperature. The samples were subsequently mixed with 2 mL of 1 M NaCl, shaken, and then left to separate overnight. The chloroform-containing organic layer at the bottom of tube was then collected from tube and was passed through a 0.2 μm PTFE filter before transferring to GC vials. The samples were analyzed using gas chromatography/mass spectrometry (Model 2010, Shimadzu, Columbia, MD) with a Restek Rtx-5MS (30 m × 0.25 mm × 0.25 μm) column with helium as the carrier gas. The inlet temperature and pressure were set at 250 °C and 14.4 psi. The total flow rate and split ratio were 46.6 mL/min and 30:1, respectively. The oven temperature was initially set at 80 °C for 1 min and then ramped up from 80 to 220 °C at a rate of 15 °C/min and held for 4 min at 220 °C. The MS ion source temperature was 250 °C with an interface temperature of 280 °C. Standards were prepared by dissolving PHB (natural origin, Sigma-Aldrich) in chloroform at a concentration of 30 mg/mL and then incubating at 105 °C for 120 min in a sealed test tube. After cooling to room temperature, the sample was then diluted to several known concentrations using chloroform, which were then analyzed under the conditions described above to generate a standard curve. A new set of standards was generated weekly.

### Single-cell protein quantification

Frozen cell pellets were thawed on ice and resuspended in 0.2 μm filter sterilized cell lysis buffer composed of 50 mM ammonium bicarbonate and 10% n-propanol in MilliQ H_2_O. Resuspended pellets were then further diluted in cell lysis buffer according to the OD600 of the culture at time of collection. Cultures with an OD600 < 4.0 were diluted 1:2 while cultures with OD600 > 4.0 were diluted 1:20. These diluted resuspensions were lysed via pressure cycling using a Barocycler 2320EXT (Pressure Biosciences, Inc, South Easton, MA, USA) with the following parameters: held pressure: 45 kpsi, on time: 30 s, off time: 10 s, cycles: 60, temperature: 37° C. SCP was quantified in the cell lysates using the Qubit protein assay, following the manufacturer’s protocol. Intracellular SCP content was calculated by normalizing the protein concentration in the lysates to the biomass of the culture at the corresponding time point.

### Amino acid composition analyses

Remaining culture (400–500 mL) at the end of incubations was centrifuged at 10,000 × g for 5 min and the supernatant was discarded. Cell pellets were frozen at −80 °C for 24 h and then dried using a lyophilizer (Labconco, Kansas City, MO). Methionine was oxidized with performic acid and hydrolyzed with Phenol/6N Hydrochloric acid in a heating block according to standard method AOAC 994.12. Tryptophan was hydrolyzed with 4.2N sodium hydroxide (alkaline hydrolysis) in a vacuum hydrolysis tube (heating block) as previously described (Dai et al. 2014). The rest of the essential amino acids were hydrolyzed in 6 N HCl vapor under vacuum (acid hydrolysis) in a microwave protein rotor (Milestone connect, Ethos up, Sorisole (BG)–Italy). All the essential amino acids were quantified in native form by scheduled Multiple Reaction Monitoring (sMRM) of the fragments after adjusted to pH of the mobile phase via SCIEX MS/MS (6500+QTRAP, Framingham MA).

### Proteome profiling by liquid chromatography tandem mass spectrometry (LC-MS/MS)

Cell lysates harvested from days 0, 1, and 7 and used for protein quantification underwent a second round of pressure cycling to facilitate peptide digestion for proteomics analysis. Briefly, 95 μL of each lysate was mixed with 5 μL of 1 μg/mL sequencing grade modified trypsin (Promega V511A, Rockford, IL) dissolved in cell lysis buffer. An additional 50 μL of cell lysis buffer was added to the mixture, increasing the total volume of each sample to 150 μL. Cell lysates were digested into peptides via pressure cycling technology (PCT) (Schultzhaus et al. 2019) using the following Barocycler parameters: Held pressure: 45 Kpsi, On time: 50 s, Off time: 10 s, Cycles: 60, Temperature: 37 °C. Peptide samples were frozen at −20 °C before drying by SpeedVac (ThermoFisher Scientific). Dried samples were then reconstituted in 50 μL of 0.1% v/v formic acid. Aliquots of 5 μL were taken and diluted again in 0.1% formic acid.

Peptide samples were injected onto a liquid chromatography–tandem mass spectrometry (LC–MS/MS) system consisting of an Ultimate 3000 LC coupled to the nanoFlex ion source of an Orbitrap Fusion Lumos Tribrid mass spectrometer (ThermoFisher Scientific, Waltham, MA), as previously described (Bird et al. 2023; Coppens et al. 2023). The Ultimate 3000 system performed nanoLC separations at a flow rate of 300 nL/min for a total of 240 min. The Orbitrap MS acquired data in OT-IT mode, by which the Orbitrap (OT) acquired precursor mass scans and the ion trap (IT) acquired product ion scans. The Orbitrap was operated in data-dependent mode with dynamic exclusion enabled. Samples were analyzed using a previously described method (Coppens et al. 2023).

Mass spectrometry data were searched by Proteome Discoverer (or PD, version 3.2.0.450, ThermoFisher Scientific). In PD, spectra were recalibrated with the Spectrum Files RC node. Recalibrated spectra were then searched with the Mascot search engine using the following parameters: 2 maximum missed cleavages, precursor mass tolerance of ± 10 ppm, fragment mass tolerance of ± 0.6 Da, Carbamidomethyl (C) as a static modification, and Deamidated (N,Q), Oxidation (M), and Acetyl (Protein *N*-terminus) as dynamic modifications. Peptide identifications were subsequently validated by the Percolator node using q-value based validation. To enable label-free quantification (LFQ), spectra were analyzed with the Minora Feature Detector node. Feature alignment and quantification were then performed by the Feature Mapper and Precursor Ion Quantifier nodes, respectively. The Top N Average method (*N*=3) was used for protein abundance calculations. The final consensus report containing quantified protein abundances was exported from Proteome Discoverer and analyzed with the *tidyproteomics* package (Jones et al. 2023) in R (version 4.4.2). In *tidyproteomics*, the randomforest method was automatically selected based on the package’s ranking criteria. Missing values were then imputed using the base R min function and column method in *tidyproteomics*. An absolute log_2_ foldchange of 2 and two-tailed Student’s *t*-test *p*-value < 0.05 (Johnston 1970) were used as cutoffs for all expression analyses of individual proteins. Statistical significances in differentially enriched Gene Ontology (GO) terms (Ashburner et al. 2000; The Gene Ontology Consortium et al. 2023) were calculated using the Wilcoxon sign-ranked test (Wilcoxon 1945). The Kyoto Encyclopedia of Genes and Genomes (KEGG) Database and BioCyc Genome Database Collection (Kanehisa et al. 2016; Karp et al. 2019) were used to curate lists of proteins involved in metabolic pathways of interest for further downstream analyses. Protein Accession IDs from the UniProt Knowledgebase (The UniProt Consortium 2025) were referenced when discussing specific proteins in text. All mass spectrometry data have been made available in the PRIDE data repository via ProteomeXchange accession PXD064900 (see Data Availability).

## Results

### Biomass production in response to different gas ratios

**Fig. 1:**
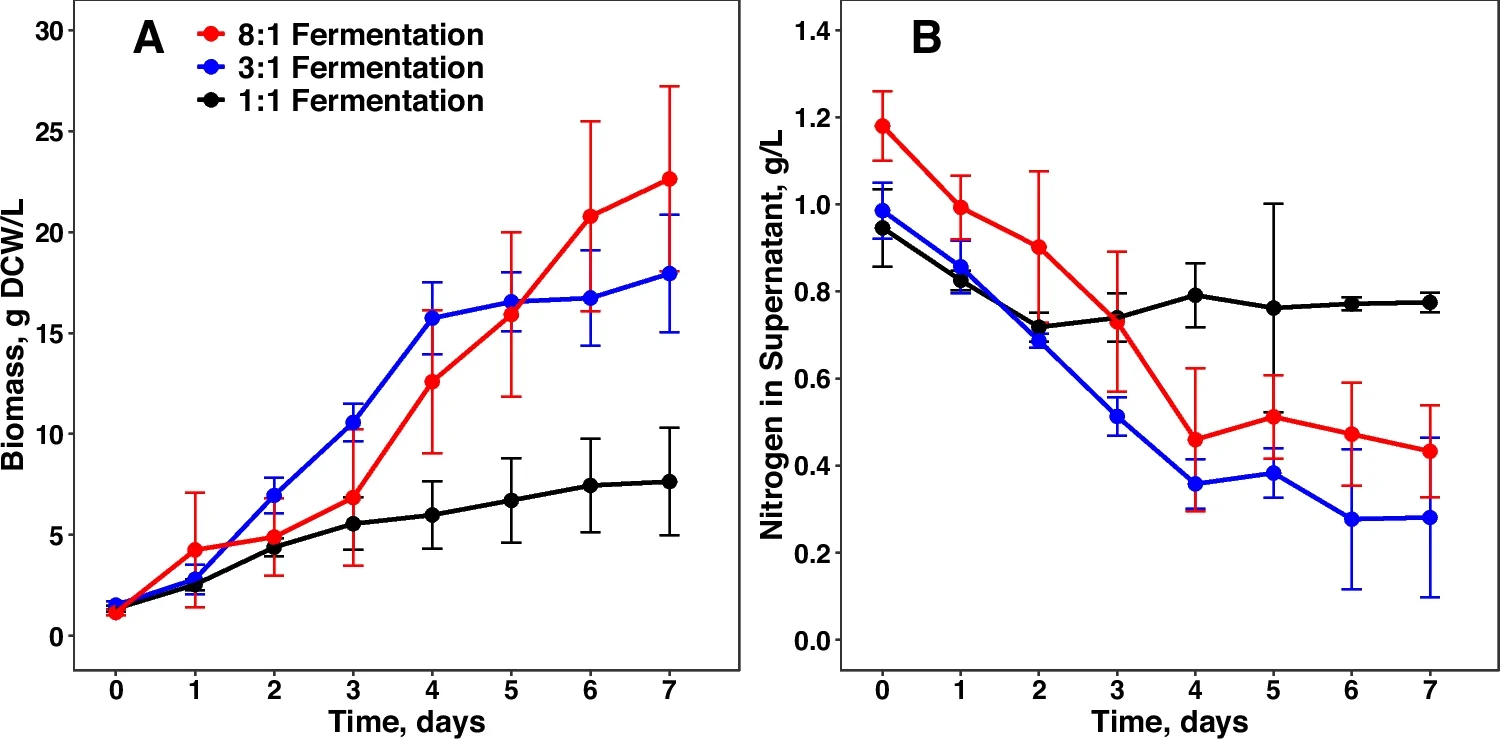
Biomass production (**A**) and nitrogen consumption (**B**) by *Cupriavidus necator* H16 in response to the three different H_2_:CO_2_ ratios used during gas fermentation experiments. Data points represent the mean ± 1 standard deviation of biological triplicates

We conducted fermentations with three different ratios of H_2_ and CO_2_ in the inlet gas stream (8:1, 3:1, or 1:1 H_2_:CO_2_) to assess whether different proportions of supplied gas substrates would influence fermentation productivity (Fig. 1). Growth between the 8:1 and 3:1 fermentations was statistically similar over the week-long incubations, with biomass respectively peaking at 22 ± 5 and 18 ± 3 g DCW/L by day 7 (Student’s *t*-test *p*-value = 0.29). By comparison, we observed significantly less growth using the 1:1 H_2_:CO_2_ ratio, with a maximum biomass concentration of 8 ± 3 g/L after seven days, with *p*-values of 0.006 and 0.043 when compared to the 8:1 and 3:1 fermentations, respectively (Fig. 1a).

We measured the total nitrogen concentration that remained in the reactor supernatant at each time point (1.0 g-NH_4_-N/L was supplied) as an additional metric for growth (Fig. 1b). As expected, total nitrogen concentration in the supernatant samples decreased over the course of the week-long incubations. Total nitrogen consumption occurred at rates of 0.18 ± 0.05 and 0.16 ± 0.02 g/L/day in the 8:1 and 3:1 fermentations between days 0 and 4 respectively, after which the nitrogen consumption respectively slowed to 0.01 ± 0.05 and 0.02 ± 0.05 g/L/day. These data are consistent with biomass measurements for the 3:1 fermentation, where exponential growth slowed at day four (Fig. 1a). Total nitrogen decreased in supernatants from the 1:1 fermentations at a similar rate as the 8:1 and 3:1 fermentations until day 2 (0.11 ± 0.05 g/L/day), after which the consumption was negligible for the rest of the incubation period (Fig. 1b), likely related to the overall lower growth we observed with our biomass measurements (Fig. 1a).

### Impacts on PHB production

**Fig. 2:**
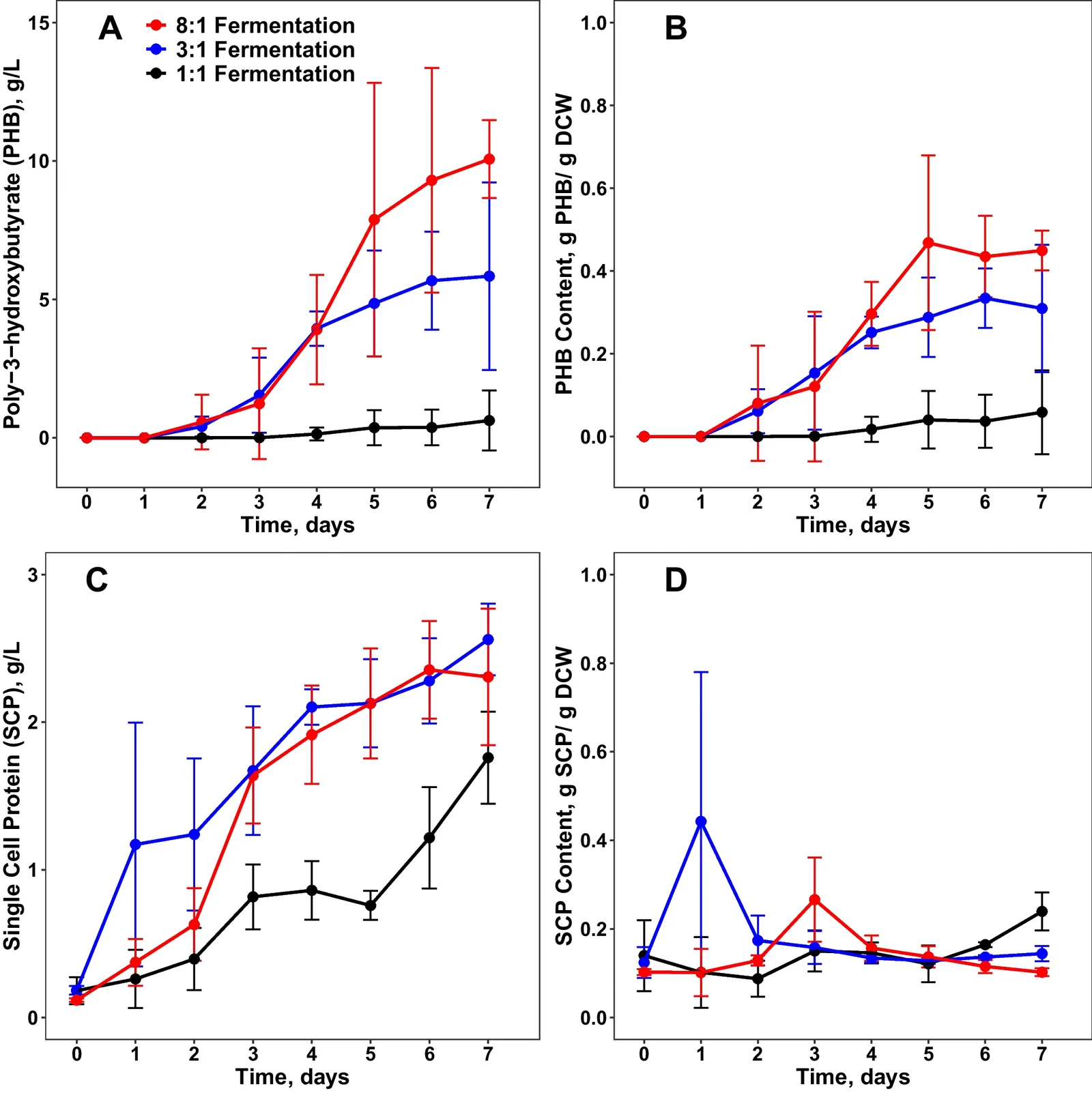
Poly-3-hydroxybutyrate (PHB) and single-cell protein (SCP) produced by *C. necator* in response to the three different H_2_:CO_2_ ratios used. Concentrations of PHB and SCP were normalized to reactor volume (panels **A** and **C**) and biomass dried cell weight (panels **B** and **D**) measured from the same sample. Data points represent the mean ± 1 standard deviation of biological triplicates

*C. necator* produced high quantities of poly-3-hydroxybutyrate (PHB) in both the 8:1 and 3:1 fermentations (Fig. 2a). PHB concentrations increased exponentially between days 2 and 5, before slowing down for the remainder of the incubations. By the end of the week-long incubations, 10.0 ± 1.4 and 5.8 ± 3.4 g/L PHB were produced in the 8:1 and 3:1 fermentations, respectively (*p*-value = 0.23). We observed large variation in PHB concentration during these fermentations, particularly at days 5 and 6 during the 8:1 fermentation and day 7 during the 3:1 fermentation. This variation may be partially explained by differences in DO saturation within individual replicates, where the demand for oxygen exceeded the ability of the controller to maintain the DO setpoint without creating explosive gas mixtures. We recorded low DO (~ 2% saturation) within one biological replicate in both the 8:1 and 3:1 fermentations during exponential growth (Online Resource 1a–b). Given that PHB accumulation has been linked to oxygen supply (Lambauer et al. 2023), it is likely that the variation in oxygen availability that we measured influenced PHB production. We normalized the PHB concentration to the DCW of biomass collected at the same time point to calculate intracellular PHB content. PHB contributed to a large proportion of *C. necator* biomass during these incubations, peaking at 0.46 ± 0.21 g PHB/g DCW on day 5 during the 8:1 fermentations and 0.33 ± 0.072 g PHB/g DCW on day 6 in the 3:1 fermentations (Fig. 2b). An order of magnitude less PHB accumulation was observed at any time point during the 1:1 fermentations by comparison, peaking at 0.36 ± 1.08 g/L on day 7, which equated to 0.059 ± 0.102 g PHB/g DCW.

### Impacts on SCP production

SCP titers increased throughout the experiment for all conditions tested, though we observed some differences in production between the different gas compositions (Fig. 2c). Similar to what we observed with overall biomass and PHB production, SCP concentrations for the 8:1 and 3:1 fermentations were comparable to each other for most of the incubation period, reaching 2.3 ± 0.5 g/L and 2.6 ± 0.2 g/L for the 8:1 and 3:1 fermentations, respectively, by day 7 (*p*-value = 0.54, Fig. 2c). On day 1, we observed large variation in SCP concentration in the 3:1 fermentation due to one of the biological replicates having a much lower concentration than the others from that time point (0.228 g/L compared to 1.78 and 1.528 g/L). This difference may be explained by the longer lag phase we observed in this particular replicate, as the biomass produced was also lower at this time point (see Online Resource 2a for biomass data from individual replicates). While we are unable to exclude this low value from the dataset, recalculating the mean SCP concentration without this specific replicate yields 1.6 ± 0.2 g/L protein, a value higher than what we observed in both the 8:1 and 1:1 fermentations at the same point during the incubation. SCP concentrations in samples taken from the 1:1 fermentations increased at a lower rate than the other two conditions for the first two days. SCP concentrations then plateaued between days three and five, before increasing again, peaking at 1.8 ± 0.3 g/L on day seven. Final SCP concentrations were similar across all three treatment groups (*p*-value = 0.25 and 0.58 for 3:1/1:1 and 8:1/1:1 comparisons, respectively).

Overall, SCP content within the biomass was comparable between all three gas ratios utilized, consistently measuring between 0.1 and 0.2 g SCP/g DCW at all time points over the course of the incubation period, aside from higher values on day 1 in the 3:1 fermentations, day 3 in the 8:1 fermentations, and day 7 in the 1:1 fermentations (Fig. 2d). We did observe the highest SCP content in the 3:1 fermentations sampled at day 1, reaching 0.44 ± 0.34 g SCP/g DCW. As explained previously, the large standard deviation is due to low SCP yield from one individual replicate (see Online Resource 2e for data on individual replicates). Recalculating without this replicate results in an even higher mean protein content of 0.62 ± 0.17 g SCP/g DCW. We also observed a spike in protein content in the biomass from the 8:1 fermentations samples at day three, reaching 0.27 ± 0.10 g SCP/g DCW. Interestingly, by day seven, biomass collected from our 1:1 incubations exhibited significantly higher protein content than the other conditions, reaching 0.24 ± 0.04 g SCP/g DCW compared to 0.10 ± 0.01 and 0.14 ± 0.02 g SCP/g biomass measured in the 8:1 (*p*-value = 0.044) and 3:1 fermentations (*p*-value = 0.036), respectively.

### Essential amino acid content within SCP

**Fig. 3:**
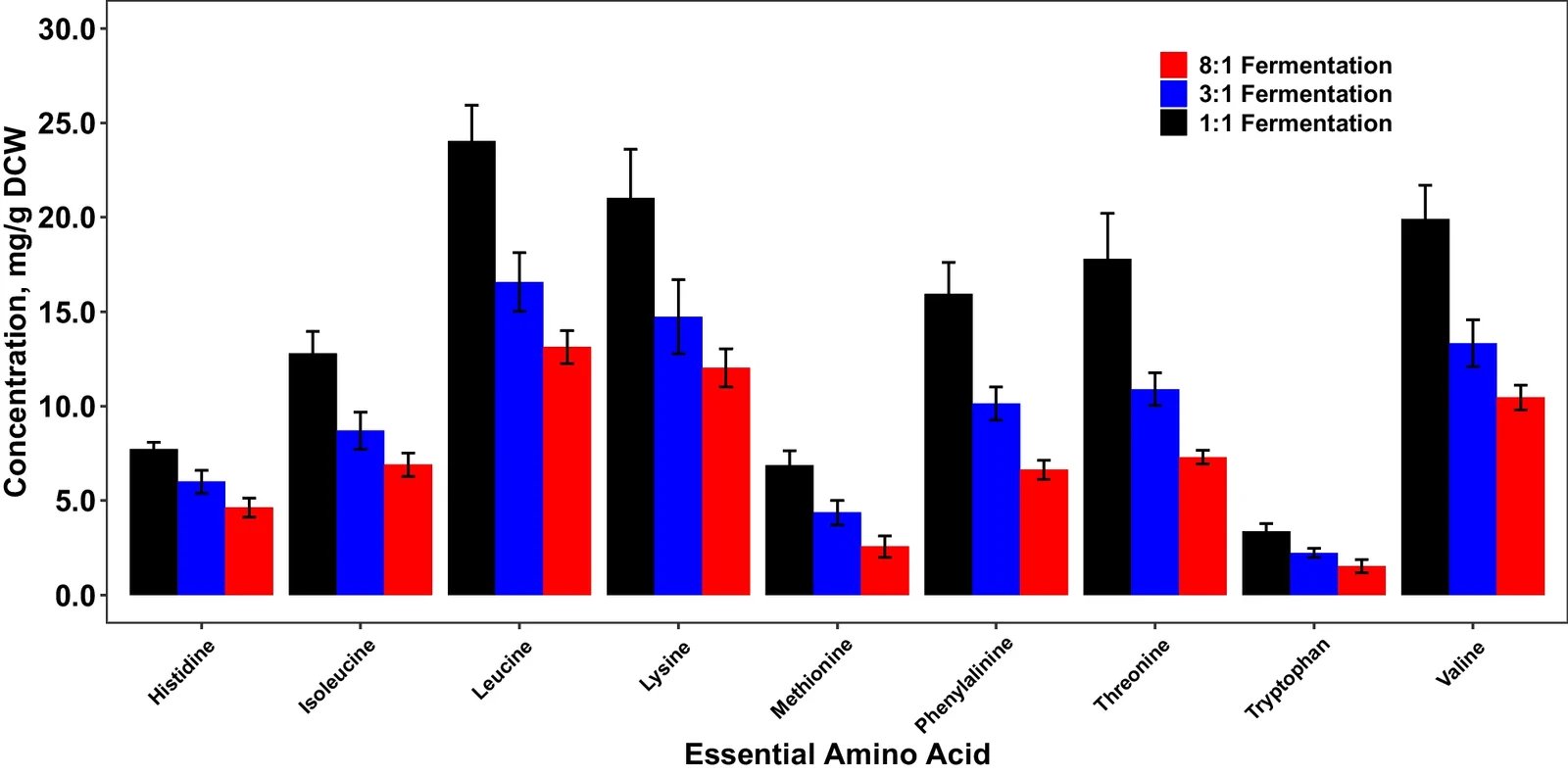
Concentration of essential amino acids (EAA) measured within *C. necator* biomass from samples collected at the end of fermentations using three different gas ratios. Data represent the mean ± 1 standard deviation of biological triplicates

Given the potential use of SCP as a nutritional resource, we quantified the presence of the 9 essential amino acids (EAAs) unable to be synthesized by humans in the *C. necator* biomass collected at the end of the incubations for all three conditions (Fig. 3). Normalizing the amount of biomass produced indicated that the 1:1 fermentations resulted in the highest yield (mg EAA/g DCW) for all EAAs, despite the overall biomass being the lowest under those conditions. Leucine and lysine were found in greatest relative proportions in biomass samples, while tryptophan was consistently found in the lowest relative proportion.

### Proteome profiling as a function of H_2_:CO_2_ ratio

**Fig. 4:**
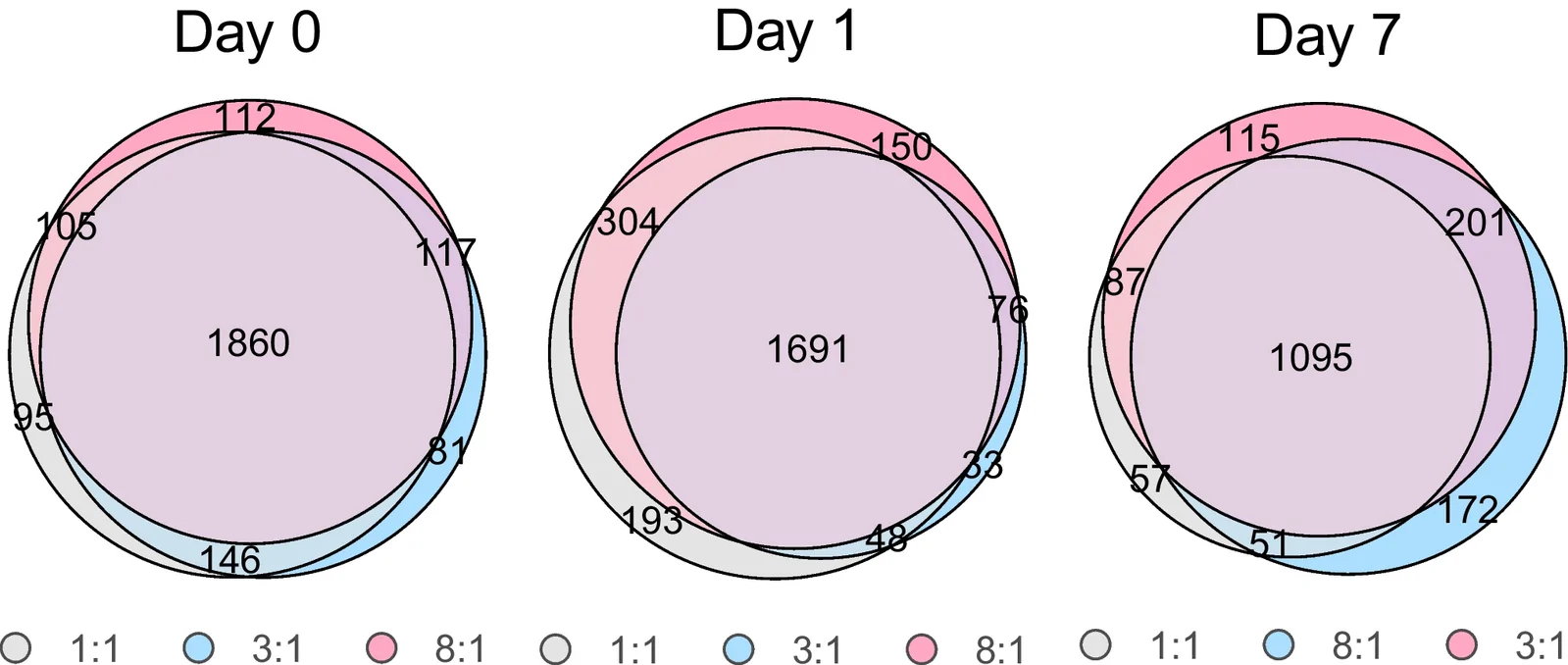
Euler diagram showing the distribution of high-confidence (confidence interval > 95%) protein identifications made by Mascot across all experimental conditions and time points analyzed. Values shown represent the average counts for biological triplicates

To gain insight into *C. necator*’s intracellular responses across different H_2_ and CO_2_ gas ratios for fermentation, we performed proteome profiling by liquid chromatography–tandem mass spectrometry (LC–MS/MS). We analyzed samples taken from days 0 and 7 to examine changes in protein expression between the beginning and end of our fermentations. As we discuss individual proteins, we will provide Uniprot protein accession numbers in parentheses. Since we observed higher intracellular protein content in samples collected from two replicates of the 3:1 fermentations on day 1 (Fig. 2d), we decided to also include samples from that time point in our analyses. We detected 2,733 unique peptide spectra across all 27 samples analyzed, representing 42% of protein-coding genes found within the genome (Little et al. 2019). When examining the presence/absence and distribution of the expressed proteins across our samples, we found that the majority of detected proteins (> 85%) were shared within samples from all three H_2_:CO_2_ ratios at days 0, 1, and 7 (Fig. 4). Given the higher mean SCP concentration and content within biomass produced in the 3:1 fermentations on day 1 (Fig. 2c and d), we investigated whether some of the unique proteins detected by proteomics could explain this phenotype. Despite the spike in mean SCP production measured at day 1 in the 3:1 fermentations compared to the other conditions (Fig. 2c and d), only 33 proteins were uniquely found in the 3:1 fermentations, compared to the 193 and 150 uniquely found in the 1:1 and 8:1 fermentations at that time point, respectively (Fig. 4). Among the proteins unique to the 3:1 fermentation samples from day 1, we detected the aerotaxis sensor receptor *B1038* (Q0K2E5) and a subunit of nitrate reductase *narH2* (Q0JYX6), potentially indicating the sensing and/or preparation for oxygen limitation (see Online Resource 8 for the complete list of proteins unique to each fermentation condition at each time point).

Using the Gene Ontology (GO) Knowledgebase (Ashburner et al. 2000; The Gene Ontology Consortium et al. 2023), we grouped the proteins according to their associated biological processes, cellular components, and molecular functions. We performed an enrichment analysis to determine whether proteins associated with particular GO terms were differentially expressed in response to the different gas ratios utilized. We note that several proteins in our dataset were not assigned GO terms, and as such were excluded from these analyses. We decided to consider the samples from the 3:1 fermentations as the experimental group for these comparison tests. When samples were grouped by biological process, we generally observed more statistically significant differences in GO term enrichment earlier in incubations (Online Resource 3). On day 0, we detected significant increases in the enrichment of proteins involved in cell organization and biogenesis, RNA metabolism or transcription, protein metabolism, cell cycle or cell proliferation, transport and other metabolic processes in 3:1 fermentations versus the 1:1 samples (hereafter referred to as the 3:1/1:1 comparison). When comparing the 3:1 fermentations to the 8:1 fermentations (referred to as the 3:1/8:1 comparison) on day 0, we detected increased enrichment in the protein metabolism category and decreased enrichment in the signal transduction and “other metabolic processes” categories. By day 7, we observed decreased enrichment only in proteins associated with protein metabolism in the 3:1/1:1 comparison and no significant differences in the 3:1/8:1 comparison. A similar trend was observed when the data were grouped by cellular component GO terms, where the number of differentially enriched ontology groups decreased with incubation time in both the 3:1/1:1 and 3:1/8:1 comparisons (Online Resource 4). There were very few significant differences in enrichment when proteins were grouped according to the molecular function GO terms (Online Resource 5).

**Fig. 5:**
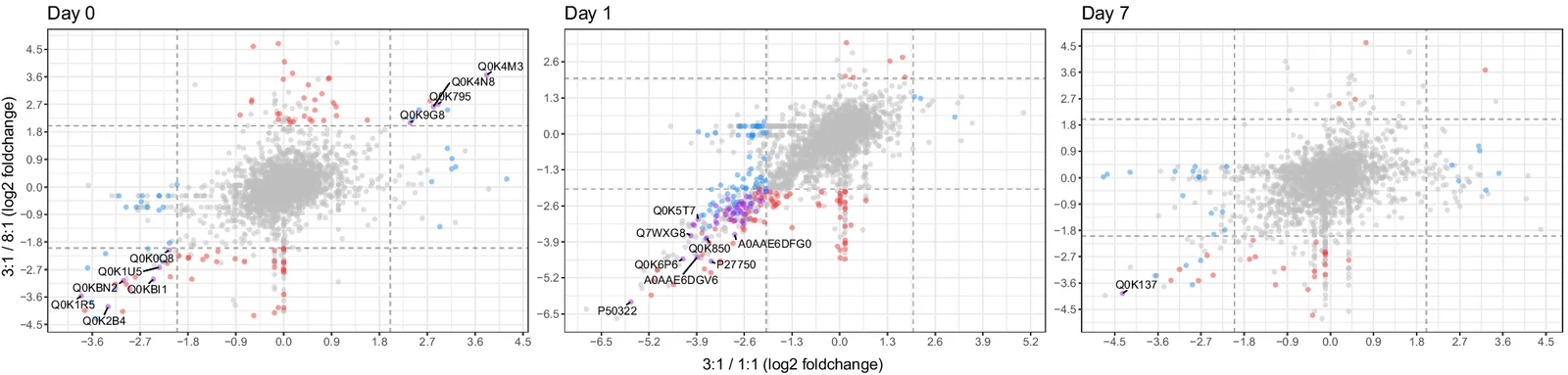
Two-way comparison of differences in protein expression between the 3:1 H_2_:CO_2_ fermentations and the other two conditions. Blue-colored points represent proteins with significant differential expression in the 3:1–1:1 comparison, while red points indicate significant differential expression in the 3:1/8:1 comparison. Proteins with significant differential expression in both the 3:1/8:1 and 3:1/1:1 comparisons are colored purple, and those whose expression was the greatest among the dataset were also labeled with their Uniprot accession numbers. Significance was determined by having an absolute log_2_ foldchange greater than 2 and Wilcoxon signed-rank test *p*-value < 0.05

We also looked for differences in expression at the individual protein level to further understand *C. necator*’s response to the three different H_2_:CO_2_ ratios (Fig. 5). As with the GO term analyses, the data from the 3:1 fermentation served as the frame of reference for these comparisons. We used both an absolute log_2_ fold change in abundance greater than 2 *and* a Student’s *t*-test *p*-value less than 0.05 as the threshold for significant differential expression. To effectively highlight the differences in protein expression that were specifically unique to the 3:1 fermentation proteomes, we plotted the expression of proteins from the 3:1/1:1 comparison against their expressions in the 3:1/8:1 comparison (refer to Online Resources 6 and 9 for details on single-way protein expression). We detected 54 proteins whose expression in fermentations was significantly lower in the 3:1 than both the 1:1 and 8:1 fermentations at all three time points, 41 of which were found in the day 1 samples. Most proteins in this group were associated with membrane transport, transcriptional regulation, and carbon metabolism (see Online Resource 10 for detailed list). A few proteins of interest include enoyl-CoA hydratase/isomerase *A2979* (Q0K7G6), used during the β-oxidation of fatty acids and intracellular poly(3-hydroxybutyrate) depolymerase *PhaZ1* (Q0KCI0), involved in the degradation of PHB polymers. Other proteins found in decreased abundances in the 3:1 fermentation on day 1 include *MetW* (Q0KF57), a regulator of methionine synthesis through activation of homoserine O-succinyltransferase *MetXS* (Q0KF58) catalytic activity (Hasebe 2021) and the plasmid-derived glyceraldehyde-3-phosphate dehydrogenase *cbbGP* (P50322), which is involved in carbon fixation via the Calvin cycle. As with day 1, most of the 10 differentially expressed proteins were involved in transport, regulation, and carbon metabolism on day 0. The only protein found differentially expressed in the 3:1 fermentations on day 7 compared to the two other conditions was an ABC transporter (Q0K137) (Online Resource 10).

### Expression of metabolic pathways of interest

We focused the rest of our analyses on the expression of proteins in metabolic pathways of interest to this study (H_2_-oxidation, carbon fixation, PHB synthesis, and EAA synthesis). Given the similarities in biomass, PHB, and SCP production between the 3:1 and 8:1 fermentations, we decided to consider the 1:1 fermentations as the reference group that would best highlight the differences in *C. necator*’s physiology with respect to these gas ratios tested. We observed changes in the expression of most proteins within every pathway that we examined as a function of both gas ratio supplied and incubation time. We recognize the difficulty in assigning statistical significance to the expression of most proteins we examined in our comparisons due to having few biological replicates for each sample group as well as the large variation that exists within these sample groups. Despite the low sample size and high variability, we did observe significant differences in the expression of several proteins within our chosen pathways of interest. We will highlight these proteins as well as general trends in pathway expression with the understanding that the differential abundances of these proteins are not solely responsible differences in *C. necator*’s physiological responses to the different H_2_:CO_2_ ratios that we observed. Rather, we believe this expression data can serve as a foundation for future analyses, where an increased number of biological replicates under varying conditions will allow for greater interrogation of *C. necator*’s physiology during gas fermentation.

**Fig. 6:**
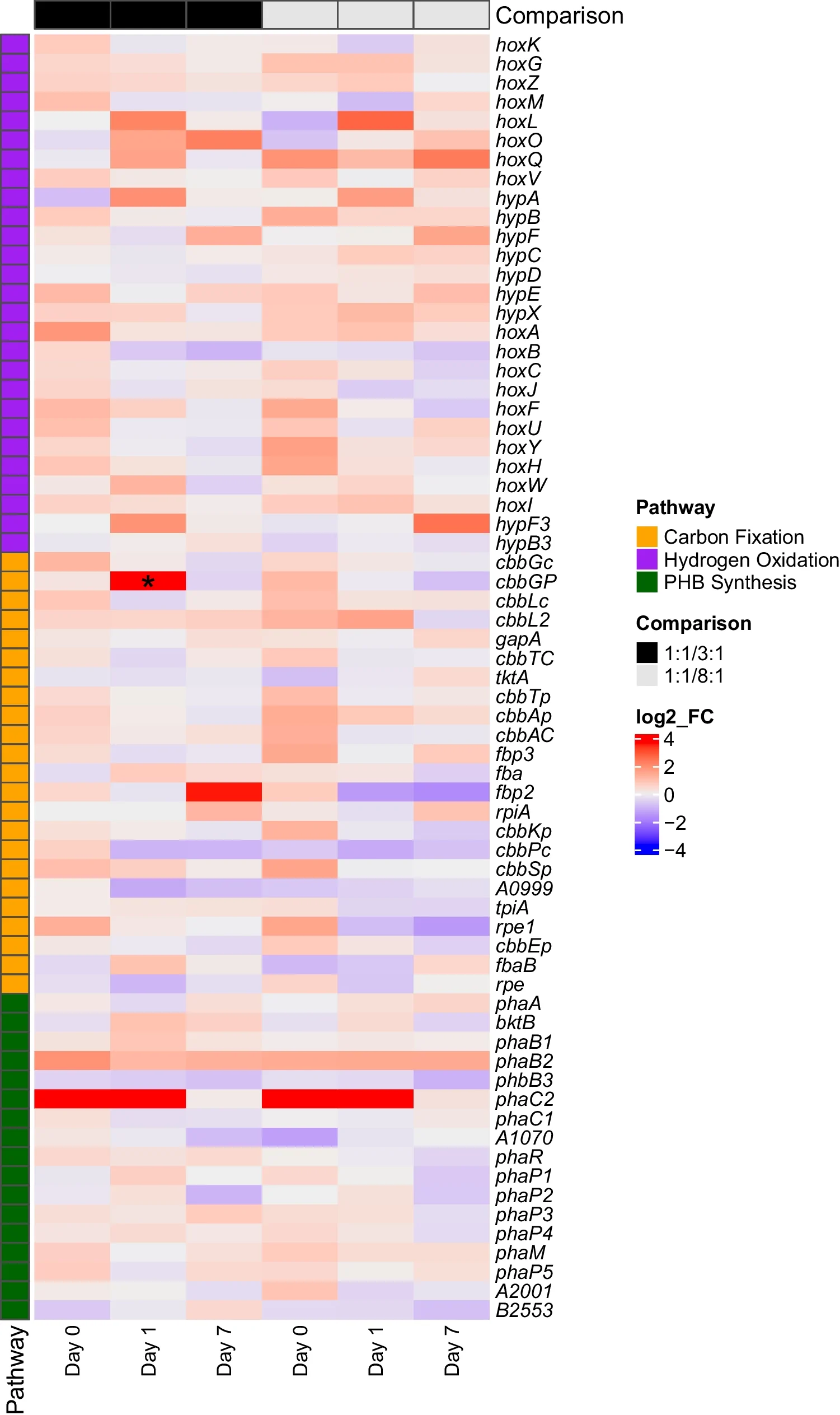
Heatmap describing differential expression analysis of proteins involved in H_2_ oxidation, carbon fixation, and the synthesis and granulation of PHB in the 3:1/1:1 (left) or 3:1/8:1 (right) comparisons. Tiles are organized by the comparison performed (1:1/3:1 or 1:1/8:1) and by metabolic pathway. Tiles marked with an asterisk represent proteins having significantly different expression in samples from the 1:1 fermentation compared the other condition (determined by having an absolute log_2_ fold change greater than 2 and a *t*-test *p*-value less than 0.05)

Proteins involved in hydrogen oxidation were not significantly differentially expressed across conditions and time according to our statistical thresholds (absolute log_2_ fold change > 2 and *t*-test *p*-value < 0.05). Nevertheless, proteins involved in formation and maturation of both the soluble hydrogenase (SH), responsible for reduction of NAD^+^ to NADH, the membrane-bound hydrogenase (MBH), which couples electron flow to the respiratory complex, as well as the regulatory hydrogenase (RH) that controls transcription of hydrogenase genes (Lenz et al. 2002), were generally upregulated (Fig. 6 and Online Resource 11) in the 1:1 fermentations compared to the other conditions early in the incubation. With respect to the actinobacterial-type hydrogenase (AH) that is also present in *C. necator*’s genome (Panich et al. 2021), we only detected 2 of 6 AH assembly proteins (*hypB3* and *hypF3*, A0AAF1D5A1 and Q7WXM8) in its proteome and neither of the two AH subunits in our dataset. The number of upregulated proteins and the extent of the upregulation of the hydrogenase-related proteins generally decreased over time in the 1:1/3:1 comparison. By day 7, the majority of these proteins were either expressed at similar levels or slightly downregulated in the 1:1/3:1 comparison. One notable exception to this was. the MBH accessory protein *hoxO* (P31910), which was upregulated in the 1:1 fermentations. With our 1:1/8:1 comparison, we noticed that both *hoxO* (P31910) and another MBH accessory protein *hoxL* (A0AAF1D564) had the lowest log_2_ fold change at day 0. On day 1, MBH accessory protein *hoxM* (A0AAF1D563), MBH subunit *hoxK* (A0AAF1D561), and RH subunit *hoxJ* (Q7WXU0) were detected in lower abundances in samples from the 1:1 fermentations. By day 7, RH subunits *hoxB* (Q79IP7) and *hoxC* (Q79IP6), as well as SH subunit *hoxF* (A0AAF1D567) were found in decreased abundance in the 1:1 fermentations (Fig. 6 and Online Resource 11).

With respect to carbon fixation, we detected all proteins found in both the chromosomal and plasmid-derived copies of the Calvin cycle (Fig. 6 and Online Resource 12). The expression of most proteins in this pathway was higher in the 1:1 fermentations compared to both the 3:1 and 8:1 fermentations on day 0. With the exception of the class 2 fructose-1,6-bisphosphatase *fpb2* (P19911) in the 1:1/3:1 comparison, expression generally decreased over time, where expression of most in the 1:1 fermentations was either similar or lower than the other two conditions by day 7. The plasmid-derived glyceraldehyde-3-phosphate dehydrogenase *cbbGP* (P50322) was the only carbon fixation protein whose average expression was significantly different in the 1:1 fermentation, where it was found in higher abundance compared to the 3:1 fermentation on day 1 (Fig. 6 and Online Resource 12).

The expression of PHB synthesis and granulation proteins also generally decreased with time in the 1:1 fermentations and to a greater extent when compared to the 8:1 fermentations (Fig. 6). We observed higher average expression of acetyl-CoA reductase *phaB2* (Q0KA69) at all time points and PHA synthase *phaC2* (Q0KA68) on days 0 and 1 in the 1:1 fermentations (Fig. 6 and Online Resource 13). Interestingly, expression of acetoacetyl-CoA synthases *phaB1* (P14697) and *phaB2* (Q0KA69) was higher at all 3 time points in both the 1:1/3:1 and 1:1/8:1 comparisons while the expression of the third acetoacetyl-CoA synthase *phaB3* (A0AAE6DI03) was lower in the same samples (Fig. 6 and Online Resource 13).

**Fig. 7:**
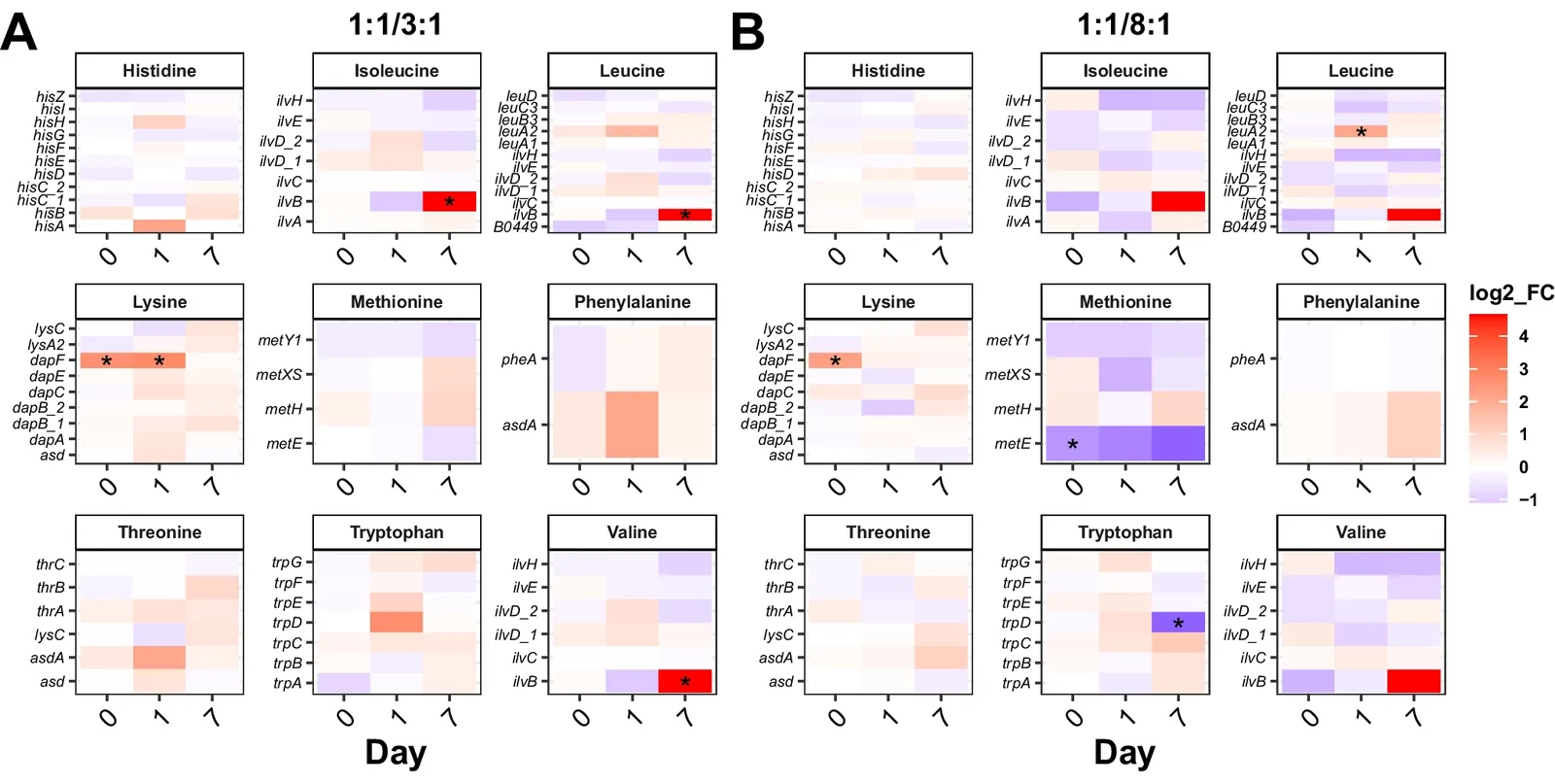
Heatmaps describing differential expression analysis of proteins involved in synthesis of essential amino acids (EAAs) in either the 1:1/3:1 (panel **A**) or 1:1/8:1 comparison (panel **B**). Tiles marked with an asterisk represent proteins with significantly different expression in samples from the 1:1 fermentation compared to the other condition (determined by having an absolute log_2_ fold change greater than 2 and a *t*-test *p*-value less than 0.05)

Several proteins involved in the synthesis of EAAs within SCP were significantly differentially expressed in our comparisons (Fig. 7). The expression of the diaminopimelate epimerase *dapF* (Q0KF42), used in lysine biosynthesis, was higher in the 1:1/3:1 comparison on days 0 and 1. In addition, the acetohydroxyacid synthase catalytic subunit *ilvB* (A0AAE6DFF6), used in the biosynthetic pathway for leucine, isoleucine, and valine was found in higher abundance in the 1:1/3:1 comparison on day 7 (Fig. 7a and Online Resources 14). We also found that the abundance of *dapF* (Q0KF42) was significantly higher in the 1:1/8:1 comparison at day 0. Expression of 2-isopropylmalate synthase *leuA2* (Q0K537), involved in the synthesis of leucine, was also significantly higher in the 1:1/8:1 comparison at day 1. Proteins with significantly decreased expression in the 1:1/8:1 comparison include 5-methyltetrahydropteroyltriglutamate–homocysteine S-methyltransferase *metE* (Q0K0V6) on day 0, and anthranilate phosphoribosyltransferase *trpD* (A0AAE6DI03) on day 7, involved in methionine and tryptophan biosynthesis, respectively (Fig. 7b and Online Resources 14).

## Discussion

Changing the ratios of H_2_ and CO_2_ gases in the substrate supply influenced *C. necator*’s growth and ability to produce PHB, SCP, and the EAAs that comprised the SCP within cellular biomass. One potential explanation for this is the C:N ratio that resulted from each gas ratio, an experimental parameter that has been shown to influence the extent to whether PHB or SCP are major components of *C. necator* biomass (Morlino et al. 2023; Yang et al. 2021). Despite not specifically quantifying dissolved CO_2_ in our bioreactors, it is possible that we indirectly adjusted C:N ratios by changing CO_2_ availability via differences in the gas flow rates between conditions and keeping starting NH_4_-N concentrations consistent at ~1.0 g/L. Nitrogen likely was not the limiting resource in our fermentations, as total nitrogen in the supernatant never fully depleted under any of our conditions (Fig. 1b). When considering H_2_ as *C. necator*’s energy source, fermentations where H_2_ was flowing at higher rates likely possessed more dissolved H_2_ available for oxidation by *C. necator*. Our growth data support this idea, as biomass production was roughly equivalent between the 8:1 and 3:1 fermentations (Fig. 1a). The 1:1 fermentations resulted in the lowest overall biomass production compared to the other two conditions, which identifies a lower bound on the system where it is likely that dissolved H_2_ was a limiting substrate. These results are similar to what was observed recently in serum bottle experiments, where a H_2_:CO_2_ ratio of 1.2:1 yielded significantly less growth than when H_2_ was supplied in higher proportions (Santin et al. 2024). While our proteomics data do suggest that different H_2_:CO_2_ ratios can exert influence on the expression of proteins related to hydrogen oxidation and carbon fixation (Fig. 6), differences in production we observed are more likely explained by differences in the relative availability of hydrogen during the fermentation process. With less available H_2_ in the 1:1 fermentations, there is less reducing power available for *C. necator* to fix carbon, leading to lower overall biomass production.

PHB has the potential to contribute significantly to the composition of *C. necator*’s biomass and is usually synthesized when carbon is available in excess and other nutrients, including nitrogen, are limiting (Morlino et al. 2023). We began to see large quantities of PHB produced during the second half of our incubations using the 8:1 and 3:1 gas ratios (Fig. 2a), where the highest PHB accumulation occurred at the same time that the rates of nitrogen consumption slowed in both our 8:1 and 3:1 fermentations (approximately day 4, Fig. 1b). Given that CO_2_ was supplied constantly throughout the entire incubation period, it was expected that nitrogen would eventually become a more limiting resource as more biomass was produced. Interestingly, the lowest mean concentrations of nitrogen that we measured were 0.28 ± 0.18 g/L after seven days in the 3:1 fermentations. This concentration may not necessarily be low enough to induce growth limitation, as previous work using another autotrophic *C. necator* strain supplied with either 0.26 or 1.0 g/L nitrogen reported similar growth dynamics at both concentrations (Yang et al. 2021). That study did not, however, examine PHB production at either nitrogen concentration. It is possible that other components within the minimal growth medium were depleted before nitrogen could become the limiting factor for growth. While phosphate limitation has been shown to induce PHB accumulation (Santin et al. 2024), phosphate was provided in excess in our medium and was likely not responsible for stimulating PHB production in our experiments. We observed very little PHB production at any point during our 1:1 fermentations, further supporting the aforementioned idea that dissolved H_2_ itself may have been the primary factor in determining whether PHB is synthesized, restricting the reducing power available to convert CO_2_ to PHB. This idea is also substantiated in our proteomics data, where general decreases in expression of PHB synthesis and granulation proteins in the 1:1 fermentations over time suggest that there was not much need to synthesize PHB when H_2_ availability is low (Fig. 6).

As expected, SCP production closely mirrored the production of biomass. Since our seed cultures were grown using fructose prior to inoculation in our bioreactors, we initially hypothesized that the increased SCP production could be attributed to the synthesis of proteins required for autotrophic growth. However, prior work has shown that *C. necator* constitutively expresses the genes involved in carbon fixation via the Calvin Cycle during heterotrophic growth (Brigham et al. 2012; et al. 2024; Hernández–Herreros; Jahn et al. 2021; Peplinski et al. 2010; Shimizu et al. 2013). When we specifically examined the expression of the Calvin Cycle proteins in our dataset, the plasmid-derived glyceraldehyde-3-phosphate dehydrogenase *cbbGP* (P50322) was the only protein whose expression was differentially significant in the 1:1 fermentation, where it was found in higher abundance on day 1 compared to the 3:1 fermentation. Expression of all other Calvin Cycle proteins in the 1:1 fermentations largely decreased over time compared to the other conditions, especially in the 1:1/8:1 comparison (Fig. 7 and Online Resource 12), also suggestive of *C. necator*’s restriction on carbon fixation due to limitations imposed by low H_2_ availability.

While SCP concentrations were statistically indistinguishable between the 8:1 and 3:1 fermentations on day 1, the average concentration was higher using the 3:1 H_2_:CO_2_ ratio, despite both fermentation conditions beginning exponential growth at similar times (Figs. 1 and 2). The higher proportion of CO_2_ in the gas stream in the 3:1 fermentations may exert some influence over SCP production earlier in incubation, whereas after the first few days, *C. necator* was able to acclimate to growth in the bioreactor setting regardless of the gas ratio provided. The low SCP concentrations and intracellular protein content measured in samples from our 1:1 fermentations are also likely related to H_2_ being the limiting resource under these conditions, given the linear growth profile we observed during these fermentations. This further indicates that SCP production is sensitive to the ratio of H_2_ provided to the system during fermentation. While we could not assign statistical significance to differential expression of hydrogenase synthesis and maturation pathways across our treatment groups (Fig. 7), the trends that we did observe in the expression of the hydrogenase subunits, accessory proteins, and maturation factors likely represent attempts to optimize electron transport capabilities and intracellular redox state in response to different H_2_:CO_2_ ratios. This idea is further substantiated by significant differential expression of other proteins involved in electron transport and/or maintaining intracellular redox homeostasis, including c-type cytochromes (Q0JYS2, Q0K5T7), cbb_3_ cytochrome oxidase subunits 1 (Q0KES7) and 3 (Q0K9A8), cytochrome bo_3_ ubiquinol oxidase subunit 3 (Q0KCQ7), thioredoxin (Q0K1R8), NAD(P)/FAD-dependent oxidoreductases (Q0K852, Q0KAU4), NADH:flavin oxidoreductase (Q0K241), and NADH-quinone oxidoreductase subunit B (A0AAE6DFG0) across comparisons and time points (Online Resources 9 and 10). Relatedly, the aerotaxis sensor *B1038* (Q0K2E5) was uniquely found in the 3:1 fermentations on days 0 and 1 (Online Resource 8), which has also been previously found in high abundances in autotrophically grown *C. necator* cultures (Kohlmann et al. 2011). This receptor is related to *E. coli*’s aerotaxis receptor *Aer*, which is responsible for monitoring changes in redox activity in the electron transport system (Kohlmann et al. 2011).

SCP consistently contributed between 10 and 25% of the biomass in *C. necator* cells during our incubations and was highest in the 3:1 fermentations on day 1 and highest in the 8:1 fermentations on day 3 (Fig. 2d). Generally, our intracellular SCP content measurements were two to four times lower than what has been reported in other recent work analyzing autotrophically grown *C. necator* biomass (Lee et al. 2025; Li et al. 2024; Yang et al. 2021), despite our high biomass yields. As discussed earlier, relatively large proportions (up to 67%) of *C. necator* biomass could be attributed to PHB accumulation (Fig. 2b), which can partially explain our low intracellular SCP content. When considering the accumulation of both SCP and PHB, we can account for a maximum of 90% of the total intracellular biomass in our data, depending on H_2_:CO_2_ ratio used and time of sampling. Interestingly, removal of the PHB fraction from our biomass calculations (Online Resource 7a) only marginally increases our intracellular SCP content within the residual cell biomass (Online Resource 7b), suggesting that our fluorescence-based protein quantification methods are an underestimation of the true values. Other quantification methods used in recent work report higher SCP concentrations within *C. necator* biomass by either measuring protein concentration directly, using colorimetric methods (Yang et al. 2021), or indirectly by calculating total nitrogen via either the Dumas method (Ismail et al. 2024) or the total Kjeldahl nitrogen method (Dou et al. 2019), and multiplying the total nitrogen concentration by the widely used conversion factor of 6.25 to estimate SCP concentration. Given mean nitrogen consumption after seven days by *C. necator* in our experiments was 0.75, 0.71, and 0.17 g/L for 8:1, 3:1, and 1:1 fermentations, respectively (Fig. 1b), we would expect to measure a maximum of 4.7 (204% higher), 4.4 (71% higher), and 1.1 (62% lower) g/L SCP in those fermentations if we then applied the 6.25 conversion factor. These calculated values would correspond to SCP contents of 37%, 36%, and 15% of PHB-subtracted residual biomass for the 8:1, 3:1, and 1:1 fermentations, respectively. These values are still approximately 50% or less of what has been reported previously. The discrepancies between our actual concentrations and theoretical maximums given the observed nitrogen consumption underscore the importance of methodology choice when quantifying SCP. Any remaining biomass is attributable to other cellular components such nucleic acids, lipids, and carbohydrates, which have been shown to contribute approximately 25% in another strain of autotrophically grown *C. necator* (Volova & Barashkov 2010). During the analysis of the cell extracts with GC–MS, we did notice the presence of several unidentified peaks that did not align with the PHB standards we used for quantification (data not shown), indicating that we have yet accounted for every compound found within *C. necator*’s biomass.

Upon normalizing EAA content to cell biomass, we observed a trend where higher proportions of H_2_ in our gas stream led to lower EAA concentrations per g DCW. This trend was consistent for all nine essential amino acids examined (Fig. 3). Our EAA concentrations per unit of biomass are lower than (but within an order of magnitude of) what has been previously reported from autotrophically grown *C. necator* cultures (Jiang et al. 2022; Lee et al. 2025; Li et al. 2024; Volova & Barashkov 2010; Yang et al. 2021). These differences are likely related to the low overall SCP content we measured in our study across all conditions at most time points, as discussed above. In addition, all of our EAA measurements were taken using samples collected at the end of the week-long incubation periods and thus only provide a snapshot view of the amino acid profile in those fermentations. It is possible that the proportions of essential amino acids within *C. necator* biomass vary as a function of the culture growth stage at the time of harvest. To our knowledge, no studies have investigated the extent to which EAA composition can change over time during gas fermentation. Our proteomics dataset did reveal significantly higher expression of several proteins involved in the synthesis of lysine, isoleucine, leucine, and valine in the 1:1 fermentations compared to the other two conditions (Fig. 7 and Online Resources 14). The decreased expression of proteins involved in methionine and tryptophan biosynthesis that we observed in the 1:1–8:1 comparison did not correlate with the higher concentration of these AAs within 1:1 fermentation biomass that we measured.

## Conclusion

Microbial gas fermentation is a promising alternative as a less resource-intensive approach for the capture and conversion of C1 compounds for manufacturing technologies, compared to some thermochemical processes. While the metabolisms of gas fermenting microorganisms are relatively flexible compared to abiotic synthesis methods, partially due to their tolerance of diverse gaseous substrates, it is important to understand the effects the composition of those gas streams can have on the physiology and productivity of the organism in question. Here, we have shown that the composition of H_2_ and CO_2_ in the gas stream supplied to *Cupriavidus necator* H16 during incubations in bioreactors influences the organism’s growth and ability to synthesize both PHB and SCP, and that the availability of dissolved hydrogen in the substrate supply likely exerts a substantial influence on fermentation performance due to its effects on reaction stoichiometry. Differences in the availability of H_2_ in the substrate supply also influenced the expression of proteins involved in the metabolic pathways of interest (H_2_ oxidation, carbon fixation, and PHB/EAA biosynthesis), and additional proteomics studies using larger sample sizes would aid in more closely aligning the biochemical responses to substrate supply with fermentation outcomes. In addition, future work further optimizing the fermentation process may further improve *C. necator* as a host for biomanufacturing. For example, the operation under elevated pressures to increase gas solubility may result in higher yields of PHB and/or SCP while an improved reactor design capable of continuous-flow operation could be implemented to maintain viability at the stage of growth where peak productivity is observed. The use of strains genetically engineered to affect PHB production pathways could be used to dictate carbon allocation away from or toward PHB synthesis within *C. necator* biomass.

## Supplementary Information

Below is the link to the electronic supplementary material.

ESM 1 (PDF 2.48 MB)

ESM 2 (XLS 129 KBX)

## Data Availability

The mass spectrometry proteomics data have been deposited to the ProteomeXchange Consortium via the PRIDE partner repository (Perez-Riverol et al., 2025) with the dataset identifier PXD064900 and https://doi.org/10.6019/PXD064900 (https://www.ebi.ac.uk/pride/archive).
