# Supplementary material for: Influences of gaseous substrate composition on aerobic gas fermentation by Cupriavidus necator
Source: Appl Microbiol Biotechnol. 2026 Aug 12;110:340. doi: 10.1007/s00253-026-13990-z (PMC13748122; doi:10.1007/s00253-026-13990-z)
Supplement: Supplementary file 1 [file 253_2026_13990_MOESM1_ESM.pdf]

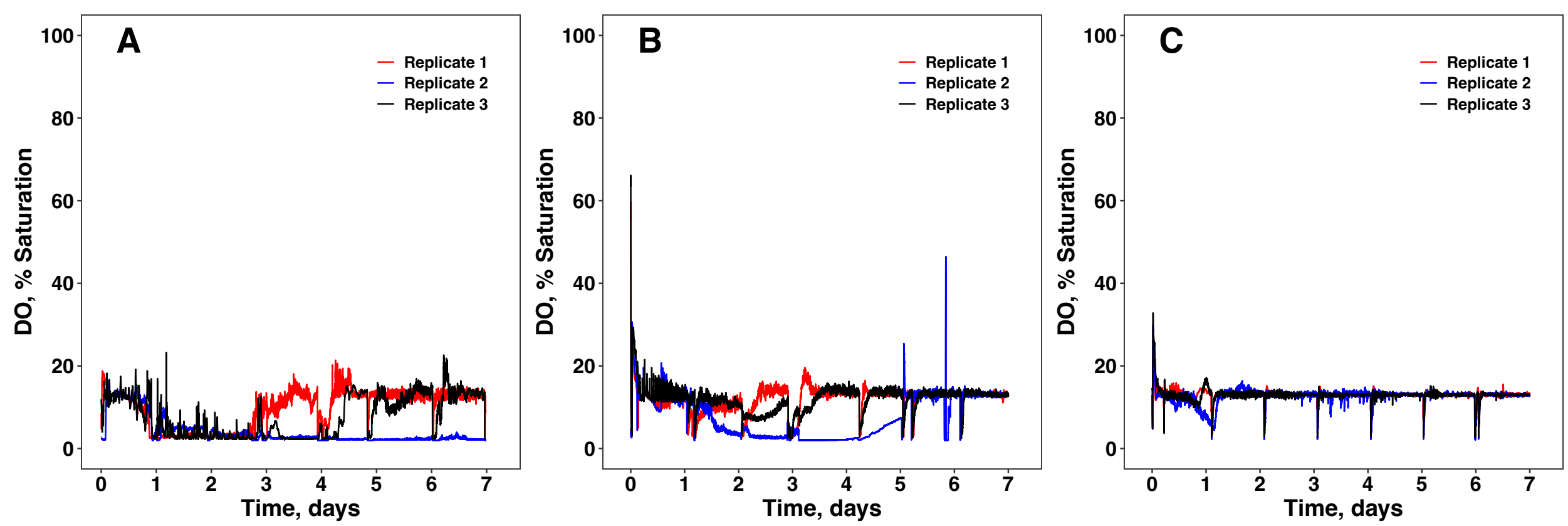

Online Resource 1. Dissolved Oxygen (DO) measurements within reactors during fermentation using the 8:1 (A), 3:1 (B), and 1:1 (C) H_2:CO_2 ratios.

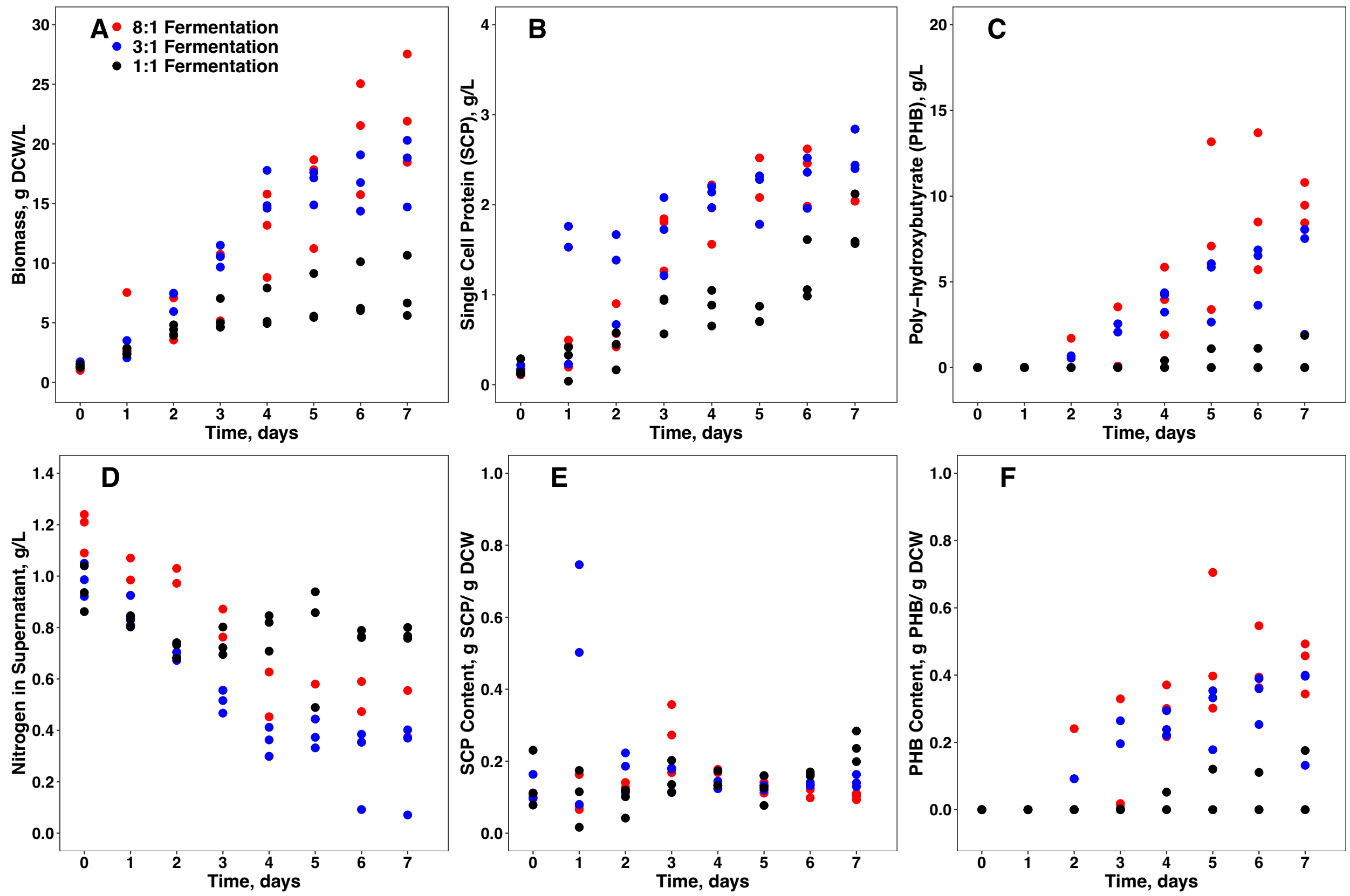

Online Resource 2. Biomass (A), Single cell protein (B), and poly-3-hydroxybutyrate (C) production as well as nitrogen consumption (D) by *Cupriavidus necator* H16 in response to the three different H_2:CO_2 ratios used. Concentrations of SCP and PHB were normalized to reactor volume (Panels B and C) and biomass dried cell weight (Panels E and F) measured from the same sample. All data points are plotted individually.

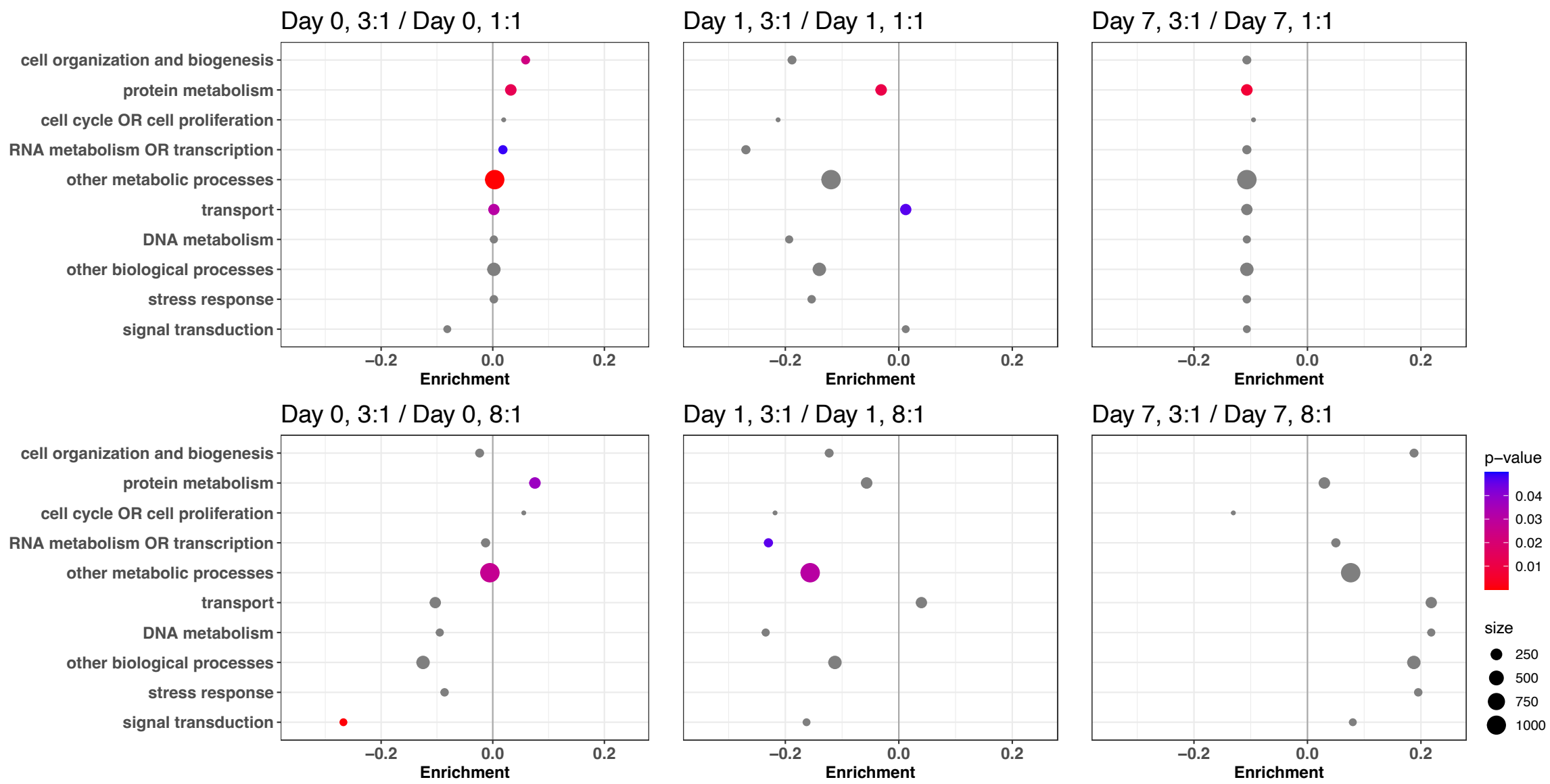

Online Resource 3 Enrichment analysis of biological process Gene Ontology (GO) terms across the time points analyzed. Data represent the mean $\log_2$ fold change in enrichment of a particular biological process between the different conditions. Comparisons made on each individual panel utilize the 3:1 gas ratio as the baseline. Data points are sized according to the number of proteins assigned to the GO term and colored according to their statistical significance. Greyed-out points indicated no difference in enrichment in the comparison made (Wilcoxon-signed rank test p-value > 0.05) while points colored along the red-blue gradient indicate significant differences in enrichment between comparisons made (p-value <0.05).

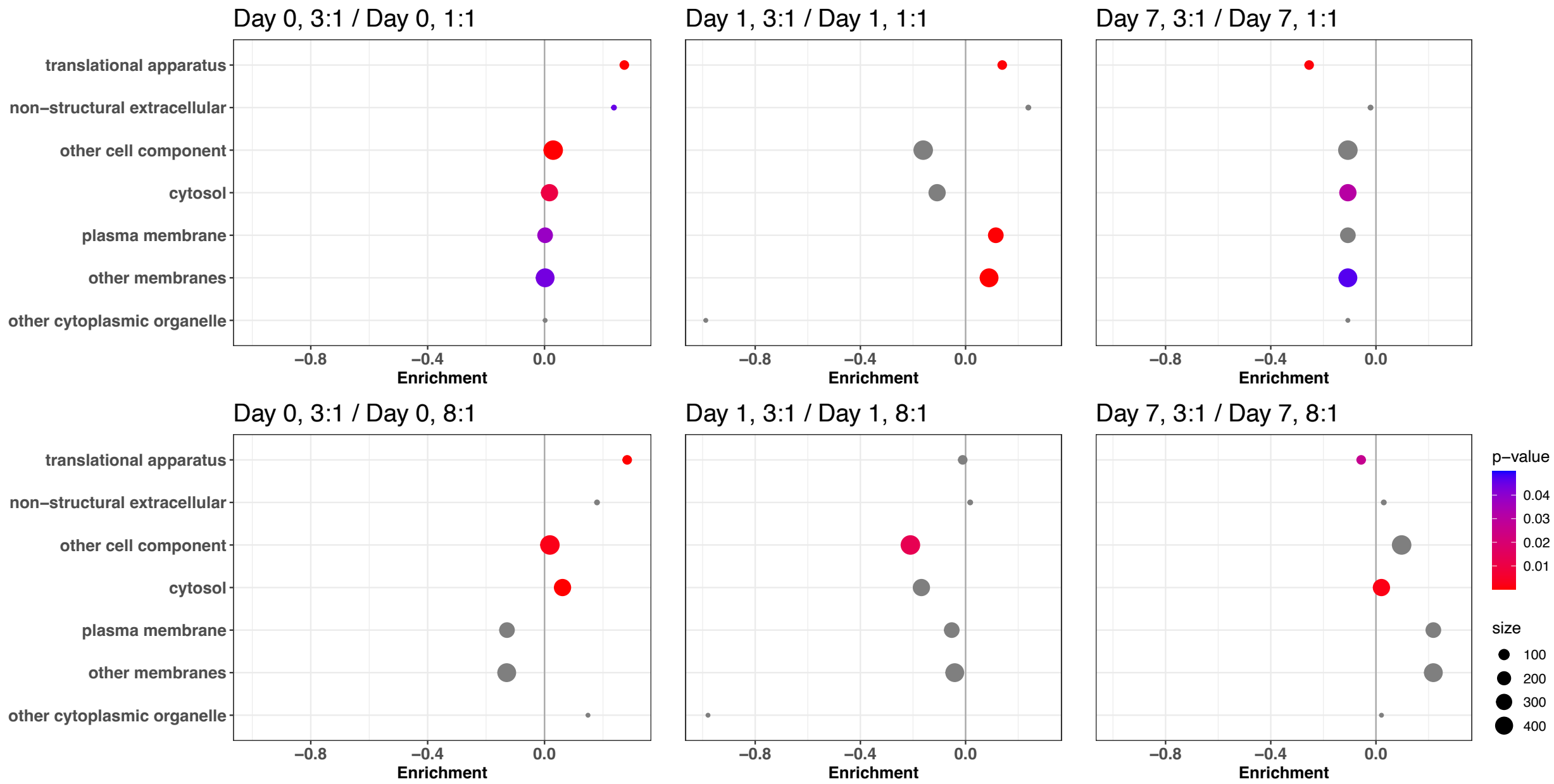

Online Resource 4. Enrichment analysis of Cellular Component Gene Ontology (GO) terms across the time points analyzed. Data represent the mean $\log_2$ fold change in enrichment of a particular biological process between the different conditions. Comparisons made on each individual panel utilize the 3:1 gas ratio as the baseline. Data points are sized according to the number of proteins assigned to the GO term and colored according to their statistical significance. Greyed-out points indicated no difference in enrichment in the comparison made (Wilcoxon-signed rank test p-value > 0.05) while points colored along the red-blue gradient indicate significant differences in enrichment between comparisons made (p-value <0.05).

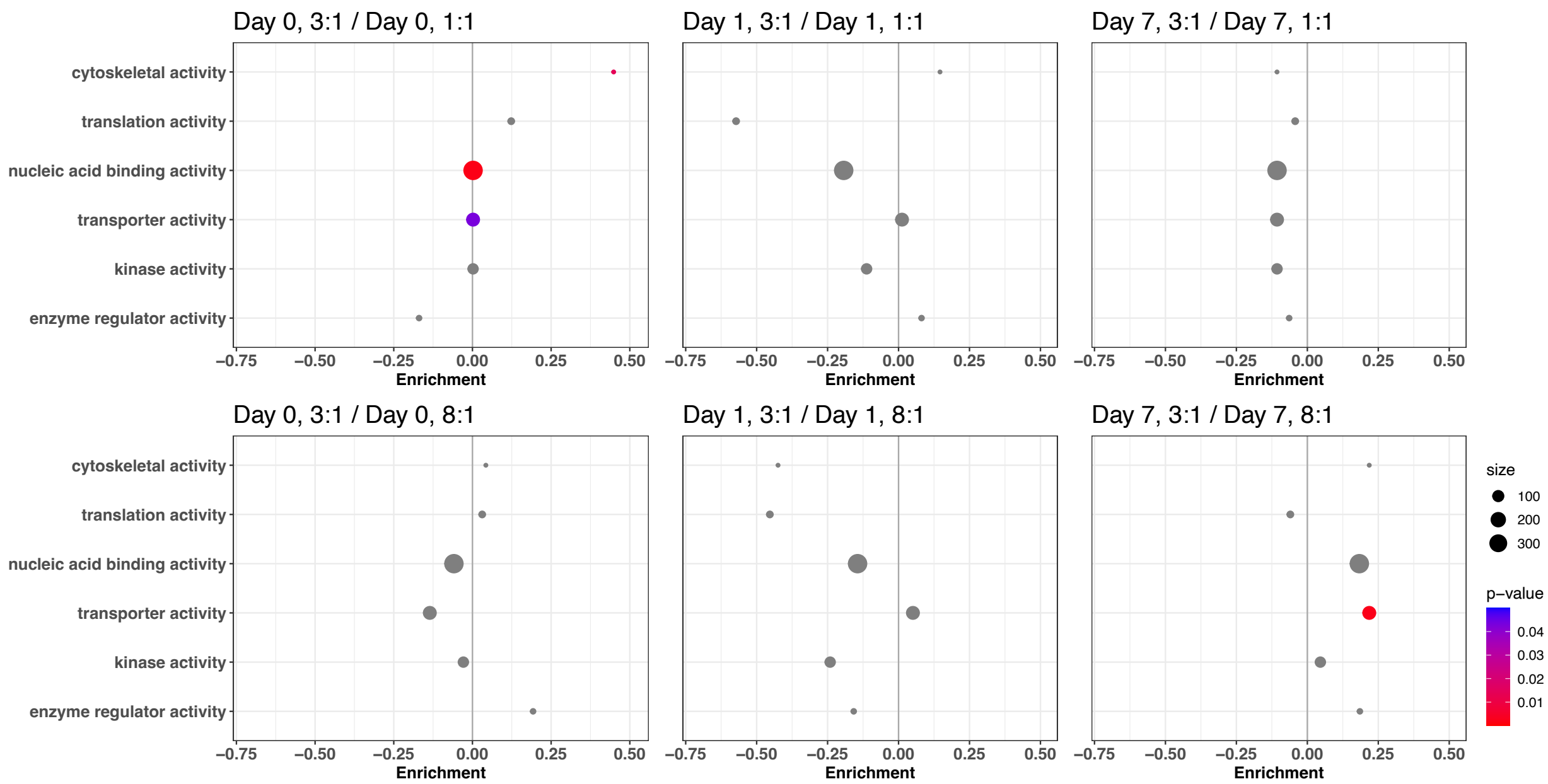

Online Resource 5. Enrichment analysis of Molecular Function Gene Ontology (GO) terms across the time points analyzed. Data represent the mean $\log_2$ fold change in enrichment of a particular biological process between the different conditions. Comparisons made on each individual panel utilize the 3:1 gas ratio as the baseline. Data points are sized according to the number of proteins assigned to the GO term and colored according to their statistical significance. Greyed-out points indicated no difference in enrichment in the comparison made (Wilcoxon-signed rank test p-value > 0.05) while points colored along the red-blue gradient indicate significant differences in enrichment between comparisons made (p-value <0.05).

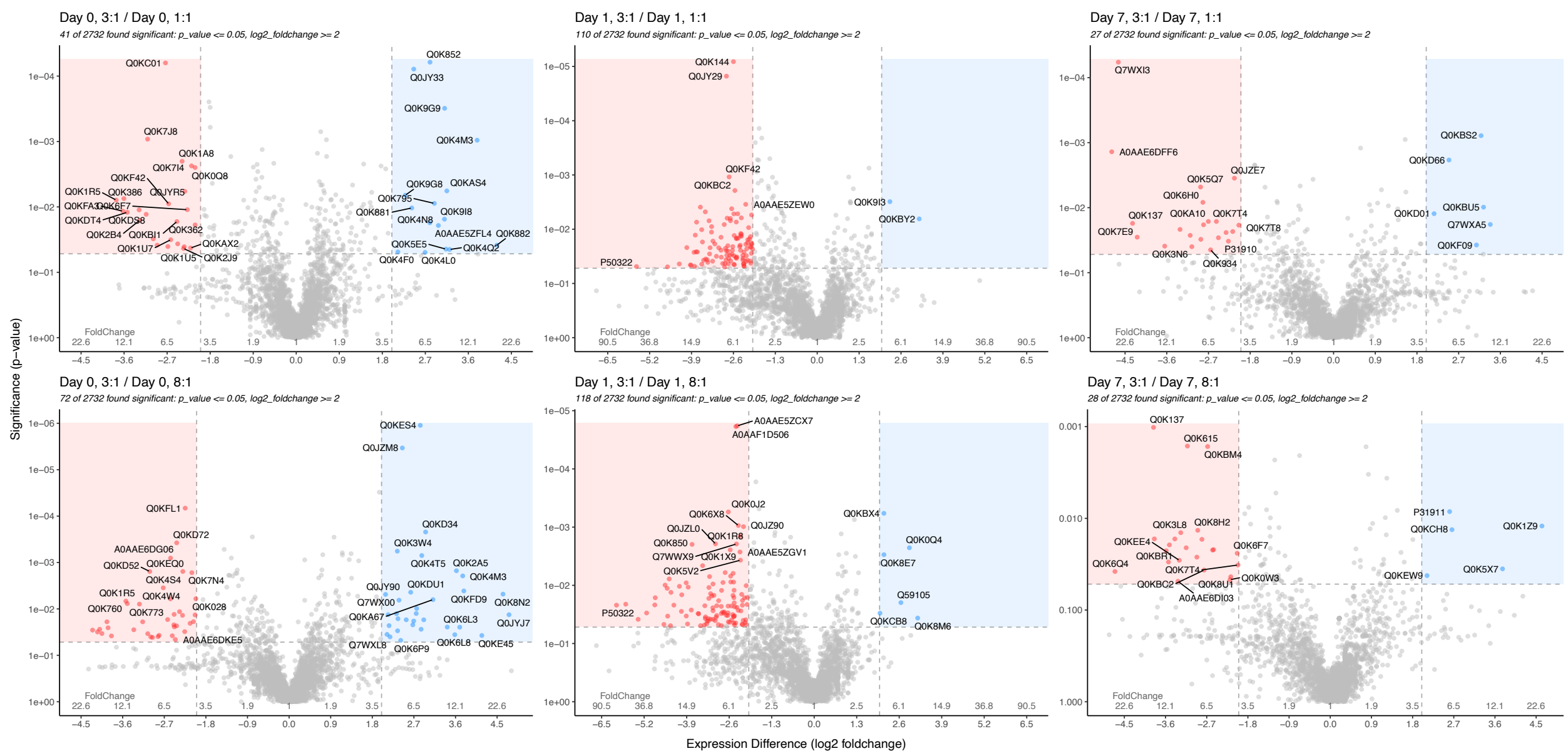

Online Resource 6. Volcano plots highlighting individual expression relative to the 3:1 H_2:CO_2 fermentations. Colored points indicate proteins whose expression is significantly higher (blue) or lower (red) in the 3:1 fermentations compared to the condition mentioned in each panel. Significance was determined by having an absolute $\log_2$ foldchange greater than 2 and Wilcoxon signed rank test p-value <0.05.

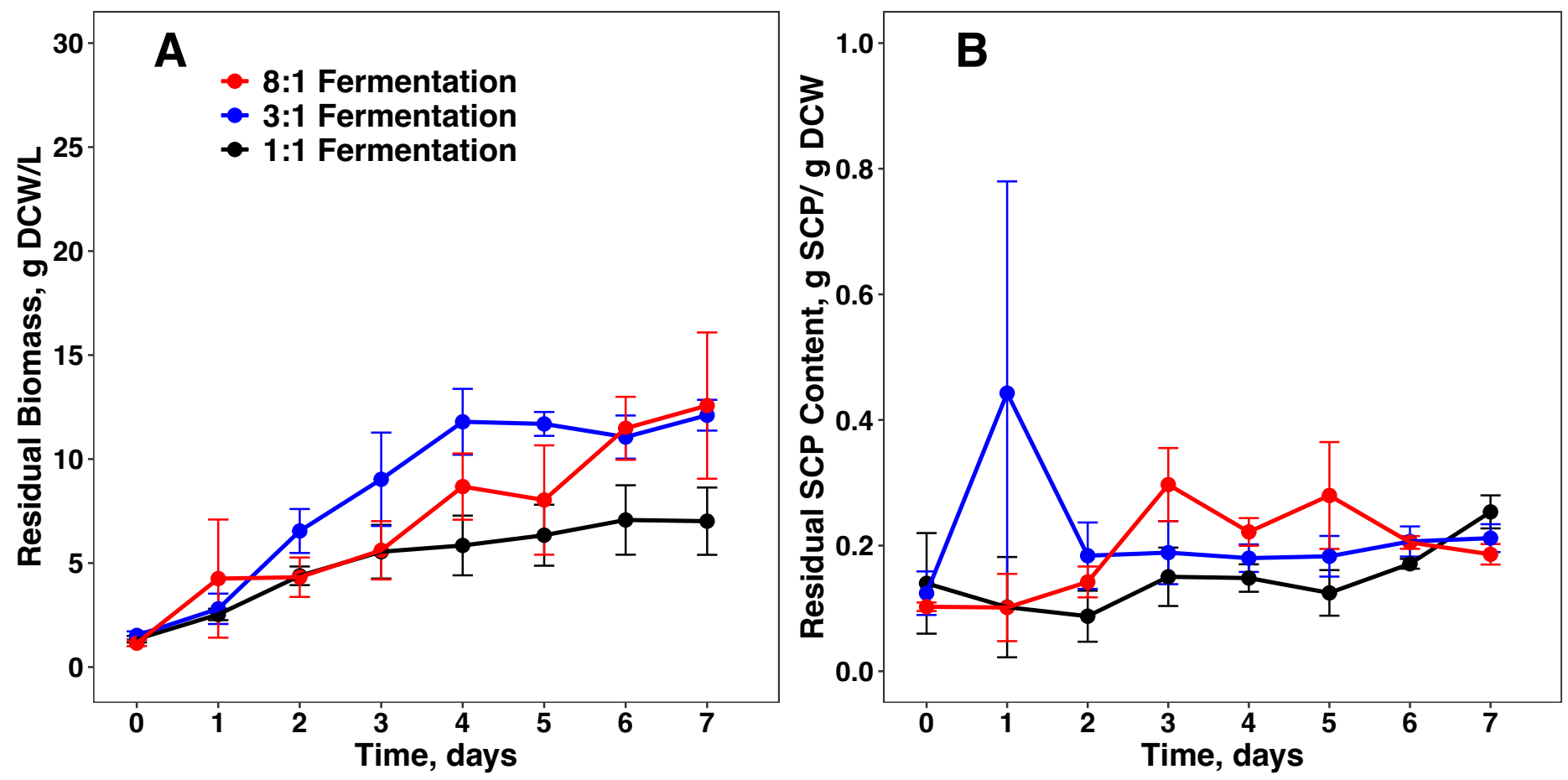

Online Resource 7. Residual biomass DCW (A) and SCP content (B) representing calculations normalized to the PHB-free portion of C. necator biomass from the three conditions. Data points represent the mean ± 1 standard deviation of biological triplicates.
